# A Case of Fracture of the Cervical Spine

**Published:** 1880-09

**Authors:** Oscar J. Coskery

**Affiliations:** Prof. Surgery, College of Physicians and Surgeons, Baltimore, Md.


### ARTICLE III.

### A CASE OF FRACTURE OF THE CERVICAL SPINE. By OSCAR J. COSKERY, M.D., Prof. Surgery, College of Physicians and Surgeons, Baltimore, Md.

In the early part of June, 1880, there was brought into the City Hospital, Baltimore, Martin B., aged 19, a powerfully made Swede, who, in tarring the stay rope, fell to the deck of the vessel from a height of thirty-five feet, striking upon his head and shoulders. He was immediately gone to by his mates and was found completely insensible. The following was his condition at time of admission into hospital: Great swelling and ecchymosis of right upper eyelid, sub-conjunctival ecchymosis of right eyeball, *supra meridian*, a puffy swelling over right temporal region, a compound fracture of left thigh and several minor bruises and scratches upon the surface generally. The heat of skin was found to be very great, the thermometer indicating 105° and 106° in the axilla. The pulse was 110, respirations 15 and very slightly stertorous, and there was twitching of the depressor muscles of the lower jaw. The penis was markedly erected, standing at right angles to the body, and if pressed downwards, at once resuming the erect state when relieved from pressure. It was also retracted and measured one inch in length. The patient became more and more comatose, and died about eight hours after receiving the injury. The diagnosis made was: fracture of the anterior fossa of the base of the skull, with fracture of the spine in the lower cervical region.

On post-mortem examination there was found a large extravasation of blood in the substance of the right temporal muscle, a considerable effusion of blood over the right hemisphere of the brain (the source of which could not be discovered), but *no* fracture of the base as had been thought.

Besides this, there was fracture of the transverse process of the seventh cervical vertebra, allowing of some compression of the cord.

The only points of interest in this case are the length of time the patient lived after so severe an injury, the small length of the penis in the marked state of priapismus, the great heat of the skin, and the fact that the penis elongated after death to nearly two inches. It also illustrated the fact insisted upon by Hutchinson that great heat of skin with priapismus is a diagnostic sign of fracture of the lower portion of the cervical spine. I should mention that on account of the evidently dying state of the man, and the difficulty of turning him over, due to the fracture of the thigh, no examination was made of his back during life.

